# 3D Bioprinted Alginate‐Gelatin‐Collagen Based Hydrogels Laden With Fibroblast and Keratinocyte Cells Show Potential for Regeneration of Injured Skin

**DOI:** 10.1002/mabi.70253

**Published:** 2026-09-09

**Authors:** Joshua Boateng, Forough Hafezi, Susan Shorter, Atabak Ghanizadeh Tabriz, Dennis Douroumis

**Affiliations:** ^1^ Faculty of Engineering and Science University of Greenwich Chatham Maritime Kent UK

**Keywords:** 3D bioprinting, alginate, collagen, fibroblasts, gelatin, keratinocytes, skin regeneration

## Abstract

Bioactive scaffolds that mimic the skin's extracellular matrix are of current scientific interest. This study developed composite biopolymer‐based bioprinted scaffolds laden with human dermal fibroblasts (HDFs) and primary epidermal keratinocytes (KCs) to generate constructs that can take active part in skin regeneration. Sodium alginate (ALG) was partially crosslinked with CaCl_2_ and combined with gelatin (GEL) and collagen (COL) to obtain composite bioinks for easy extrusion. CaCl_2_ concentration (0.2–4.5% w/v) and bioink physico‐chemical properties were optimized using rheometry and scaffolds’ stability was optimized through degradation studies. Cell viability within the printed scaffolds were evaluated (7 days) using MTT and live‐dead assays, while the stratified layers of HDFs/KCs within the scaffolds were confirmed through immunofluorescence imaging. Bioinks partially crosslinked with 0.5% w/v CaCl_2_ allowed optimal extrusion during bioprinting, while scaffolds fully crosslinked with 1.5% w/v CaCl_2_ produced scaffolds that maintained their structural integrity and cell viability with appropriate proliferation and migration over the 7 days. This is the first study comprising ALG‐GEL‐COL, laden with HDFs and KCs and bioprinted for potential skin regeneration applications following injury. The generated bioprinted scaffolds will help advance the field of tissue regeneration, including potential to use as therapeutic drug delivery platform in future studies.

## Graphical abstract

Engineered skin tissue by 3D bioprinting of hydrogels comprising alginate‐gelatin‐collagen and laden with human dermal fibroblasts and keratinocytes were successfully produced. The viability and phenotypes of the fibroblasts and keratinocytes were all well maintained within the printed constructs using MTT, live/dead and immunofluorescence assays. The engineered skin tissue constructs show potential for chronic wound healing and skin regeneration.

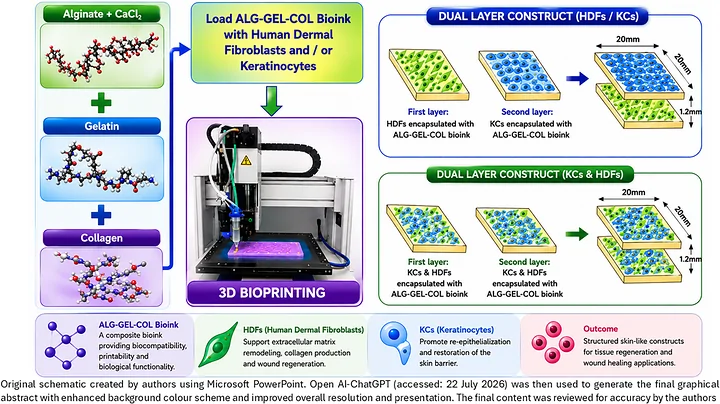

## 1 Introduction

To achieve appropriate skin regeneration following injury, it is essential to recover the stratified skin cellular structure. This is possible through maintaining cell‐cell and cell‐extracellular matrix (ECM) interactions as these are important to restore normal skin function. Conventional approaches for fabricating skin tissue constructs such as manual dispensing, freeze‐drying and particulate (porogen) leaching, do not consider the precise spatial disposition of cells within the individual strata [1, 2] and unable to fabricate constructs with complex architecture that mimic the ECM more closely [3]. Furthermore, these approaches require incubation periods of three or more weeks to obtain fully differentiated skin. Recent approaches such as 3D bioprinting offer significant advantages over conventional skin tissue engineering, allowing 3D printing of bioactive scaffolds comprising biomaterials and cells to generate living tissue constructs [4, 5, 6]. These constructs could also play a dual role as platforms for delivering various therapeutic agents such as antimicrobials [7, 8]. Biomaterials for 3D bio‐printing should possess ideal properties that ensure that cells remain viable and can differentiate. They must also be easily printable, maintain construct integrity as well as being mechanically stable after printing [9]. Embedding functional skin cells such as HDFs and KCs within the polymer ink to form a “bio‐ink” provide the opportunity to generate biologically active three‐dimensional scaffolds to aid the regeneration and repair of damaged/diseased skin tissue.

ALG is a naturally occurring, linear polysaccharide composed of guluronic and mannuronic acids [10], which is biodegradable, non‐toxic, and non‐immunogenic making it highly biocompatible. In addition, ALG is cheap and already used as a safe and biocompatible pharmaceutical excipient including as moist wound dressing [11]. As a result, ALG has been employed in tissue engineering scaffolds and 3D bioprinting systems produced via extrusion due to viscosity and fast gelation rate by forming a hydrogel through calcium and sodium ion exchange under mild temperature conditions [12, 13, 14]. The calcium crosslinked hydrogel is able to trap water and other molecules via capillary forces within the ALG matrix, whilst loaded molecules diffuse out to the target site. The above characteristics make ALG an ideal constituent for designing and fabricating 3D printing bioinks.

Though ALG presents excellent biocompatibility and mechanical stability, its poor biomimetic properties do not provide any viable cellular adhesion sites due to the absence of essential protein components. Further, the human skin cells cannot utilize ALG during ECM formation as part of the healing (regeneration) process [10, 15], therefore other biopolymers are required to modify their properties and performance in vivo. COL, which is the main component of the ECM, can be obtained from various natural sources including mammals (e.g., cows, pigs) and fish such as tilapia and cod [16, 17, 18]. It has been employed as a major component of commercially available tissue engineered scaffolds [19, 20] as well as within composite dressings for chronic wound healing applications. COL has also been used as a bioink material in 3D bioprinting either alone or in combination because of its excellent biocompatible properties [21].

However, like ALG, 3D printed COL matrices on their own possess limitations such as poor mechanical performance including brittleness, therefore bioink and printed matrix properties need to be enhanced through combination with other modifying biopolymers within a composite system. For example, cell adhesion and differentiation of ALG matrices were facilitated through the addition of GEL, which also allowed improvements in the gel viscosity [22] thus enabling more efficient extrusion during bioprinting. Blending of ALG and GEL in extrusion‐based bioprinting exploits both the thermo‐sensitive properties of GEL [23] with the ease of chemical crosslinking of ALG, allowing the design of matrices with tunable properties. Afzali and co. recently developed composite ALG, COL and hyaluronic acid‐based scaffold dressings prepared by freeze‐drying crosslinked gels (targeting ALG and hyaluronic acid for crosslinking) and demonstrated significantly improved functional properties [24] compared to dressings produced from the individual biopolymers and the corresponding non‐crosslinked composite matrices [25].

In this study, ALG partially crosslinked with CaCl_2_ was combined with GEL and COL to obtain a triple biopolymer (ALG‐GEL‐COL) composite hydrogel system to allow easy extrusion. The composite gel bioinks have been further embedded with HDFs and KCs and bioprinted to obtain cell‐laden scaffolds for potential skin regeneration. In order to achieve printability of the bioink and achieve the desired cell laden scaffold, concentration of the crosslinker and functional chemical and physical properties of the bioink were optimized by applying various analytical techniques including, rheometry, and degradation studies. Consequently, the viabilities of the cells within the bioprinted scaffolds were evaluated for up to 1 week using MTT assay and live‐dead assay via confocal microscopy. Further, the stratified layers containing HDFs and KCs within the multi‐layered ALG‐GEL‐COL scaffold were confirmed through immunofluorescence confocal imaging. To the best of our knowledge, this is the first study comprising the three biopolymers (ALG‐GEL‐COL), laden with HDFs and KCs and bioprinted for potential skin regeneration applications following injury. Somasekharan and co. prepared ALG and GEL based composite scaffolds, for bioprinting, however, the third polymer was cellulose rather than COL [26]. Bettendorf and co. bioprinted hydrogels comprising ALG, GEL and hyaluronic instead of COL for tissue regeneration. In addition, their scaffolds were laden with only KCs and no HDFs unlike the current study [27].

## 2 Materials and Methods

### 2.1 Materials

Collagen was obtained from Cell Systems, Troisdorf, Germany, and calcium chloride purchased from Honeywell, Hannover, Germany. Potassium chloride, sodium phosphate dibasic, sodium hydroxide, MTT reagent, penicillin/streptomycin, calcein, 4',6‐diamidino‐2‐phenylindole, dihydrochloride (DAPI), glycine, paraformaldehyde, Triton‐X‐100, and mounting media were all purchased from Fisher Scientific, Loughborough, UK. Potassium dihydrogen phosphate, ALG, GEL, fetal bovine serum (FBS), and dimethyl sulfoxide (DMSO) were all obtained from Sigma‐Aldrich, Dorset, UK. Goat polyclonal secondary antibody to Mouse IgG—H&L (TR), pan‐keratin (C11) mouse antibody, and fluorescein isothiocyanate (FITC) phalloidin were obtained from Abcam, Cambridge, UK. KC growth kit, dermal cell basal medium, trypsin/EDTA solution for HDFs, KCs, and Dulbecco's modified Eagle's medium (DMEM) were obtained from ATCC, Manassas, Virginia, USA.

### 2.2 Methods

#### 2.2.1 Cell Lines and Cell Culture

KCs were cultured at a temperature of 37°C with 5% CO_2_ in dermal cell basal medium that was supplemented with KC growth kit. The HDFs cells were also cultured in DMEM which was supplemented with FBS (10%) and penicillin/streptomycin (1%) at 37°C and 5% CO_2_. The co‐culturing was a 1:1 ratio of KCs to HDFs at cell concentrations of 0.5 × 10^6^ cells/mL each while incubation, and detachment were undertaken as previously reported [28]. This involved incubating the cells and changing the media every 2 days and subsequently sub‐culturing once a confluence of 80–90% was attained and the cells detached using trypsin‐EDTA based on the prescribed ATCC protocol. They were passaged (2x) every week in tissue culture flasks and discarded after 6 passages to ensure that functional cell characteristics were maintained.

#### 2.2.2 Bioink Preparation

To prepare the polymer‐based bioinks, GEL and ALG were dissolved in double deionized water to obtain concentrations of 6% w/v for each polymer. The resulting GEL‐ALG solution was then mixed with COL (10% w/v) and stirred continuously for 15 min to obtain a homogeneous composite ALG‐GEL‐COL gel mixture which was then crosslinked with different concentrations (0.2‐ 1.2% w/v) of sterile CaCl_2_. The ALG‐GEL‐COL and CaCl_2_ were mixed in the volume ratio of 4:1 to obtain a partially crosslinked bioink and subsequently sterilized by autoclaving (121°C) for at least 30 min using saturated steam under a minimum pressure of 15 psi. The effect of the autoclaving on the crosslinked composite bioinks were evaluated by analyzing the rheological properties of both the sterile and corresponding non‐sterile bioinks as detailed below. Further, a quantitative analysis for potential degradation during autoclaving was undertaken using a simple gravimetric test. The partially crosslinked bioinks were weighed (*n* = 3) before and after autoclaving and the percentage weight loss calculated as an indicator of bioink degradation.

#### 2.2.3 Rheological Characterization

The rheological properties of composite ALG‐GEL‐COL bioinks were investigated at a temperature of 37°C and the profiles compared to commercial bioink (Cellink start, CELLINK, Sweden). A MCR 702 twin drive rheometer (Anton Parr, Graz, Austria) equipped with parallel plate geometry (Anton Paar PP50, 50 mm in diameter) was used and rheological measurements done in shear thinning mode with increasing shear rates from 0.1 to 100 s^−1^ and the corresponding viscosities measured. Before the analysis, the ALG‐GEL‐COL gels were mixed with DMEM (representative of cells and bioprinting) at a bioink to medium ratio of 5:1 to ensure that the viscosity was not altered significantly.

#### 2.2.4 Bioprinting of Cell‐Laden ALG‐GEL‐COL Bioinks

To prepare cell laden bioinks containing only one type of cell (HDFs or KCs), each cell culture was separately washed with 1X PBS, trypsinized (5 min) and centrifuged at 1100 rpm (5 min). The resulting cell pellets were re‐suspended in DMEM and dermal basal medium for HDFs and KCs respectively, stained using trypan blue and the number of cells counted on a hemocytometer. Aliquots (2.4 mL) of the previously prepared ALG‐GEL‐COL (crosslinked with 0.5% w/v CaCl_2_) were placed into 3 mL BD Luer‐Lok sterile syringes (Becton Dickinson, New Jersey, USA) while 0.2 mL of the KC and HDF cell cultures were transferred into two separately labelled 1 mL BD Luer‐Lok sterile syringes (Becton Dickinson, New Jersey, USA). Cell concentration of 1×10^6^ cells/ mL for HDFs and KCs was used to prepare the respective cell‐laden ALG‐GEL‐COL bioinks for both HDFs and KCs. Effective mixing of the KC and HDF cultures and ALG‐GEL‐COL bioink was undertaken with the help of a Luer Lock Adapter (CELLINK, Gothenburg, Sweden) connecting the two syringes containing either bioink and HDF or KC together to avoid material wastage. Finally, the cell laden ALG‐GEL‐COL bioinks were transferred into sterile printing cartridges (3 mL) at 37°C for subsequent bioprinting.

**FIGURE 1:**
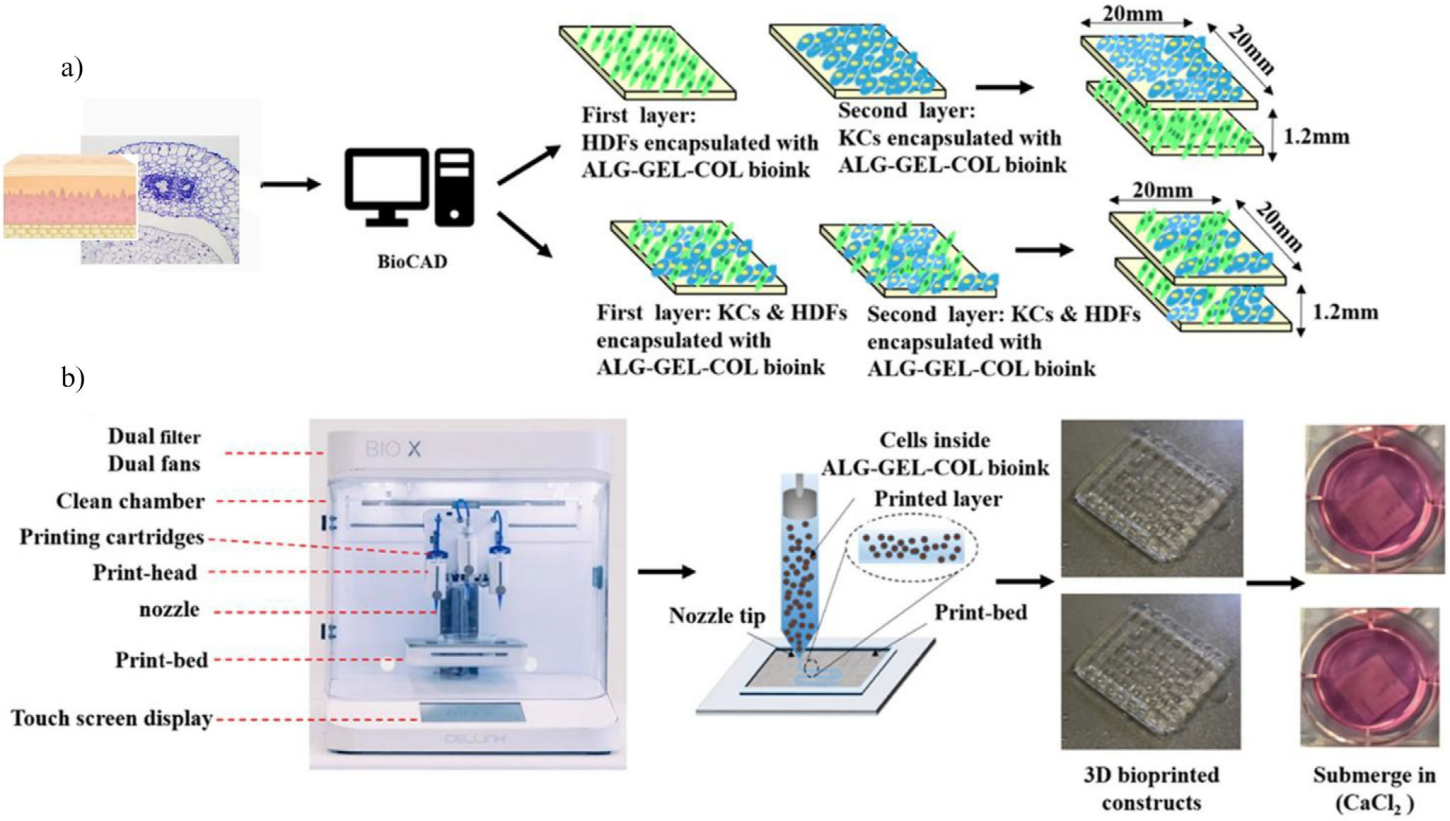
(a) Schematic Illustrating the Design of the 3D Bioprinted Skin Constructs Comprising Two Layers Having Dimensions of 20 Mm Length × 20 Mm Width × 1Mm Thickness), (b) the Working Set‐up of the BIOX Bioprinter Used in this Study and Subsequent 3D Bioprinting of ALG‐GEL‐COL Constructs Encapsulating HDFs and KCs Within both Layers.

To prepare bioinks containing both HDFs and KCs (co‐culture), the same protocol for the single cell laden bioinks was followed, however, both KCs and HDFs were first combined into a single co‐culture, centrifuged at 1100 rpm (5 min) after counting, and the cell pellet obtained re‐suspended in 1 mL of DMEM. The mixed co‐culture (0.2 mL) at concentrations of 1×10^6^ cells/ mL for both KCs and HDFs was then added to the ALG‐GEL‐COL bioink (2.4 mL), mixed thoroughly and subsequently bioprinted as described in detail below. The schematic shown in Figure 1 below summarizes the bioprinting process employed and the resultant bilayer scaffolds obtained.

#### 2.2.5 3D Bioprinting of ALG‐GEL‐COL Constructs

The bioprinting of the ALG‐GEL‐COL bioink was undertaken with the help of a BIO X (CELLINK AB, Gothenburg, Sweden) bioprinter. The bioink was first loaded into a sterile printing cartridge and printing conducted with a pneumatic based extrusion‐based print‐head. The flow of the bioink from the print‐head was controlled using speed and pressure regulators, while the shape of the scaffold was chosen from pre‐loaded scaffold designs from the BioCAD software of the BIO X bioprinter. The printability of the composite ALG‐GEL‐COL gels crosslinked with different concentrations (0.2‐1.2% w/v) of CaCl_2_ was evaluated for different printing variables including speed rate, infill density and pressure (Table S1) using a sterile high precision smooth flow tapered nozzle (Nordson‐Asymtek, Maastricht, Netherlands). A gauge of 22 G and inner diameter of 0.41 mm for ALG‐GEL‐COL was employed while a gauge and inner diameter of 20 G and 0.58 mm respectively was applied during printing of the control commercial bioink (Cellink start, CELLINK, Sweden). Prior to printing, all samples were sterilized using the printer's UV assembly while the print head was heated to 37°C. 3D constructs with dimensions of 20 mm (length) × 20 mm (width) × 1 mm (thickness) comprising two layers were bioprinted from ALG‐GEL‐COL bioinks containing (i) a co‐culture of both HDFs and KCs, (ii) HDFs only, (iii) KCs only and (iv) controls containing no cells, with the space between the two layers set at 1.2 mm. Different scaffolds from the above bioinks are as outlined below. First, 3D constructs comprising two layers from ALG‐GEL‐COL bioink loaded with mixed co‐culture of KCs and HDFs were printed and exposed to 1.5% w/v CaCl_2_ for 2 min to fully crosslink the previously partially crosslinked ALG‐GEL‐COL matrix. For the second scaffold, the first printed layer comprised 3D constructs from cell‐laden ALG‐GEL‐COL bioink containing only HDFs while the second printed layer from ALG‐GEL‐COL bioink comprised only KCs and were also exposed to 1.5% w/v CaCl_2_ for 2 min to ensure complete crosslinking of the ALG‐GEL‐COL bioink. The plain constructs (with no cells) were printed and treated the same way as the cell laden equivalents. Finally, all the bioprinted constructs were submerged in DMEM and incubated for up to 7 days at 37°C in a humidified atmosphere (5% CO_2_) and then functionally characterized as described in subsequent sections below.

#### 2.2.6 Degradation Studies

To establish the optimum concentration of the crosslinker, degradation studies were performed. Plain ALG‐GEL‐COL 3D bioprinted constructs (without cells) were submerged in CaCl_2_ at different concentrations (0.5‐4.5% w/v) for 2 min. Subsequently, CaCl_2_ was removed, and the crosslinked structures were submerged in DMEM media and kept in an incubator (37°C, 5% CO_2_) for 15 days. The structures were imaged every other day to observe their degradation behavior in the media with the media refreshed after every imaging.

#### 2.2.7 Effect of CaCl_2_ on Cell Viability of Bioprinted Constructs

To evaluate the effect of the CaCl_2_ crosslinker on the viability of both KCs and HDFs within the cell‐laden crosslinked bioinks, different concentrations of CaCl_2_ were applied over 48 h. ALG‐GEL‐COL cell‐laden bioinks were first bioprinted inside 96‐well microtiter plates using the BIO X bioprinter at concentrations of 1×10^6^ cells/ mL for both the KCs and HDFs (CELLINK AB, Gothenburg, Sweden). MTT assay was performed every 24 h over seven days with medium refreshed on the third and sixth days.

#### 2.2.8 Viability of Dispensed Cells

Cell viability within the bioprinted 3D constructs was assessed on days 1, 2, 3, 4, 5, 6 and 7. A live‐dead assay using calcein (Invitrogen, green fluorescence) to indicate viable cells and DAPI (Fisher Scientific, blue fluorescence) to depict the total number of cells was used to determine the cell viability within the 3D constructs. Each construct was washed in PBS, stained and incubated for 30 min, and washed twice with 1X PBS. A confocal microscope (Zeiss LSM 880) was used to acquire images of the cells by averaging each image 4 times. Zen Black on a 10x objective was used for image acquisition, using two tracks, set up for DAPI and calcein. Z stacks were captured, resulting in images of ALG‐GEL‐COL cell‐laden constructs having height and length of 400 and 800 µm, respectively. Three different fields were counted for each construct and the cell viability calculated using Equation 1. The 3D reconstructed images were analyzed quantitatively using 3D object counter plug‐in Image J software (1.48v) [29] to score the number of green/blue fluorescent cells embedded in the unit volume of the ALG‐GEL‐COL cell‐laden bioink [30].

(1) $$Cell\;viability\;\left(\%\right)=\frac{\mathrm{number}\;\mathrm{of}\;\mathrm{green}\;\mathrm{stained}\;\mathrm{cells}}{\mathrm{total}\;\mathrm{number}\;\mathrm{of}\;\mathrm{cells}}\;x\;100$$

#### 2.2.9 Immunofluorescence (IF)

FITC phalloidin which labels both HDFs and KCs and pan‐keratin, which labels KCs only were used to label key cellular features of both HDFs and KCs within the cell laden 3D constructs. DAPI staining was used to determine the number of nuclei and to assess overall cell morphology. The main differentiating cell label was anticipated to be keratin as KCs have an abundant source of keratin, while HDFs lack the keratin which is why pan‐keratin antibody was chosen to stain KCs (red colour). The printed bi‐layered cell‐laden composite ALG‐GEL‐COL constructs cultured for 1, 3, 5 and 7 days respectively were rinsed in 1X PBS, fixed with 4% paraformaldehyde for 30 min, and each one rinsed (3x) in 1X PBS for 5 min. The 3D constructs were then submerged in Triton‐X buffer (190 mg glycine: 100 µL Triton‐X: 50 mL PBS) for 4 min, washed (3x) with 1X PBS and left in blocking buffer (49 mL PBS: 1 mL FBS) at room temperature for 60 min. The printed constructs were subsequently exposed to primary (pan‐keratin (C11) mouse monoclonal) antibody (4:200 µL in blocking) and stored at 4°C overnight. Finally, fluorescence‐labelled secondary antibody (1:200 µL in blocking, red) (goat polyclonal secondary antibody to mouse IgG) and FTIC phalloidin (5:200 µL in blocking, green) were added. An aliquot (40 µL) of the mounting media containing DAPI was placed on a glass slide, covered with the corresponding cover slip, any excess media removed, and the cover slips sealed. Fixed 3D constructs were then visualized using the confocal microscope (Zeiss LSM 880) as described above.

#### 2.2.10 Statistical Analysis

Statistical analysis of the quantitative data was performed using one‐way ANOVA with *p* values < 0.05 considered statistically significant.

## 3 Results and Discussion

### 3.1 Rheological Analysis

**FIGURE 2:**
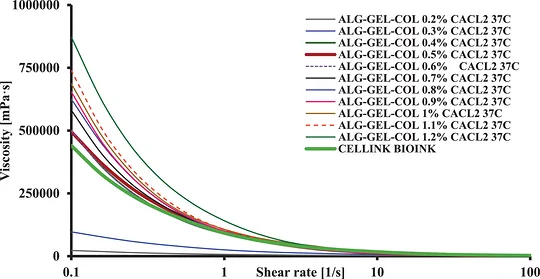
Viscosity profiles as a function of shear rate for the ALG‐GEL‐COL bioinks crosslinked with different concentrations of CaCl_2_ and compared with that of the commercial bioink at a temperature of 37°C. Data is plotted from the means of three independent readings.

Schuurman and co. analyzed the rheological profiles of cell‐laden bioinks and showed that their viscosity influenced the ability of the printed constructs to retain their shape following syringe deposition [31]. For bioprinters equipped with extrusion‐based print heads, printing fidelity usually improves at higher viscosity values which is attributed to self‐supporting capability of the bio‐ink upon deposition [32]. However, bioinks with excessively high viscosity may result in a restrictive environment for mass transport, cell proliferation and cell migration and ECM accumulation and high shear stress within the deposition nozzle leading to a higher probability of cell damage depending upon the cell type [33]. Based on this, the composite ALG‐GEL‐COL gel bioinks were crosslinked using different CaCl_2_ concentrations (0.2‐ 1.2% w/v) and their viscosities compared to the bioink supplied by the manufacturer (Cellink) to determine the CaCl_2_ concentration that provided optimum viscosity to ensure effective printing and the results are shown in Figure 2 and Table 1.

**TABLE 1:** Rheological properties of crosslinked ALG‐GEL‐COL bioinks at various concentrations of CaCl_2_ and commercial bioink 37°C (*n* = 3, ± SD).

Samples	Viscosity (mPa.s)		
		Shear rate	
	0.1 s^−1^	1 s^−1^	10 s^−1^
ALG‐GEL‐COL (0.2% w/v CaCl_2_)	22,565	7,149	2,477
ALG‐GEL‐COL (0.3% w/v CaCl_2_)	96,769	25,228	6,863
ALG‐GEL‐COL (0.4% w/v CaCl_2_)	500,134	88,910	18,235
**ALG‐GEL‐COL (0.5% w/v CaCl_2_)**	**440,123**	**92,176**	**17,162**
ALG‐GEL‐COL (0.6% w/v CaCl_2_)	495,347	94,919	13,040
ALG‐GEL‐COL (0.7% w/v CaCl_2_)	581,262	100,650	21,001
ALG‐GEL‐COL (0.8% w/v CaCl_2_)	625,733	95,103	12,269
ALG‐GEL‐COL (0.9% w/v CaCl_2_)	654,171	100,175	10,763
ALG‐GEL‐COL (1% w/v CaCl_2_)	686,659	107,066	17,875
ALG‐GEL‐COL (1.1% w/v CaCl_2_)	739,731	106,001	17,682
ALG‐GEL‐COL (1.2% w/v CaCl_2_)	872,461	141,239	15,456
Commercial bioink	**450,000**	**92,400**	**15,341**

Divalent cations such as Ca^2+^ induce rapid gelation of ALG [34] but this is dependent on their concentration. The viscosity of the ALG‐GEL‐COL bioink crosslinked with 0.2% CaCl_2_ was about 14x lower than the commercial bioink which was attributed to incomplete crosslinking arising from insufficient amount of Ca^2+^ ions. However, at of 0.5% w/v CaCl_2_ concentration, the viscosity of the crosslinked bioink was similar to that of the commercial bioink.

At this stage of the study, the primary goal was to assess the printability of the gel formulation and its ability to maintain cell viability over the specified culture period. Therefore, mechanical strength was not considered the primary focus of this preliminary investigation. However, future studies will require characterization of the mechanical properties of the printed hydrogels e.g. modulus and ultimate strength using dynamic mechanical analysis [35] to determine shape fidelity and confirm their ability to withstand normal stresses encountered when applied in vivo. However, to ensure that the partially crosslinked bioinks were not significantly degraded by the harsh autoclaving conditions, a gravimetric test was undertaken (digital photographic images of the gels before and after autoclaving are shown in Figure S1), which showed that the gels showed percentage degradation of 9.88% (± 5.4). Though not significant, this suggests that milder sterilization techniques such as UV or ethylene oxide will be a better option. Furthermore, the crosslinking density could be enhanced by slightly increasing the amount of CaCl_2_ added to the composite gels to increase their resistance to degradation to negligible values which should help maintain fidelity of the subsequent bioprinted constructs.

At a constant shear rate of 1s^−1^ the viscosity of the ALG‐GEL‐COL bioinks decreased slightly with time. For the ideal bioink (ALG‐GEL‐COL crosslinked with 0.5% w/v CaCl_2_), the viscosity decreased from 440,123 (0.1s^−1^) to 92,176 (1s^−1^) and 17,162 (10s^−1^) mPa while the commercial bioink decreased from 450,000 (0.1s^−1^) to 92,400 (1s^−1^) and 15,341 (10s^−1^) mPa respectively, showing closer alignment at all shear rates than all the other bioinks. For the other concentrations, it was observed that the structure of ALG‐GEL‐COL bioinks crosslinked with 0.2‐0.4% w/v CaCl_2_ was unstable and always collapsed during printing, whereas ALG‐GEL‐COL bioinks containing higher concentrations (0.6 – 1.2% w/v) of CaCl_2_ printed easily but required excessively higher printing pressures due to their very high viscosities. In contrast, the ALG‐GEL‐COL crosslinked with 0.5% w/v CaCl_2_ and commercial bioink were more easily printed under milder conditions and therefore ALG‐GEL‐COL crosslinked with 0.5% w/v CaCl_2_ was selected as the optimum bioink for further investigations.

### 3.2 3D Printing of Cell‐Laden Constructs Using ALG‐GEL‐COL

**FIGURE 3:**
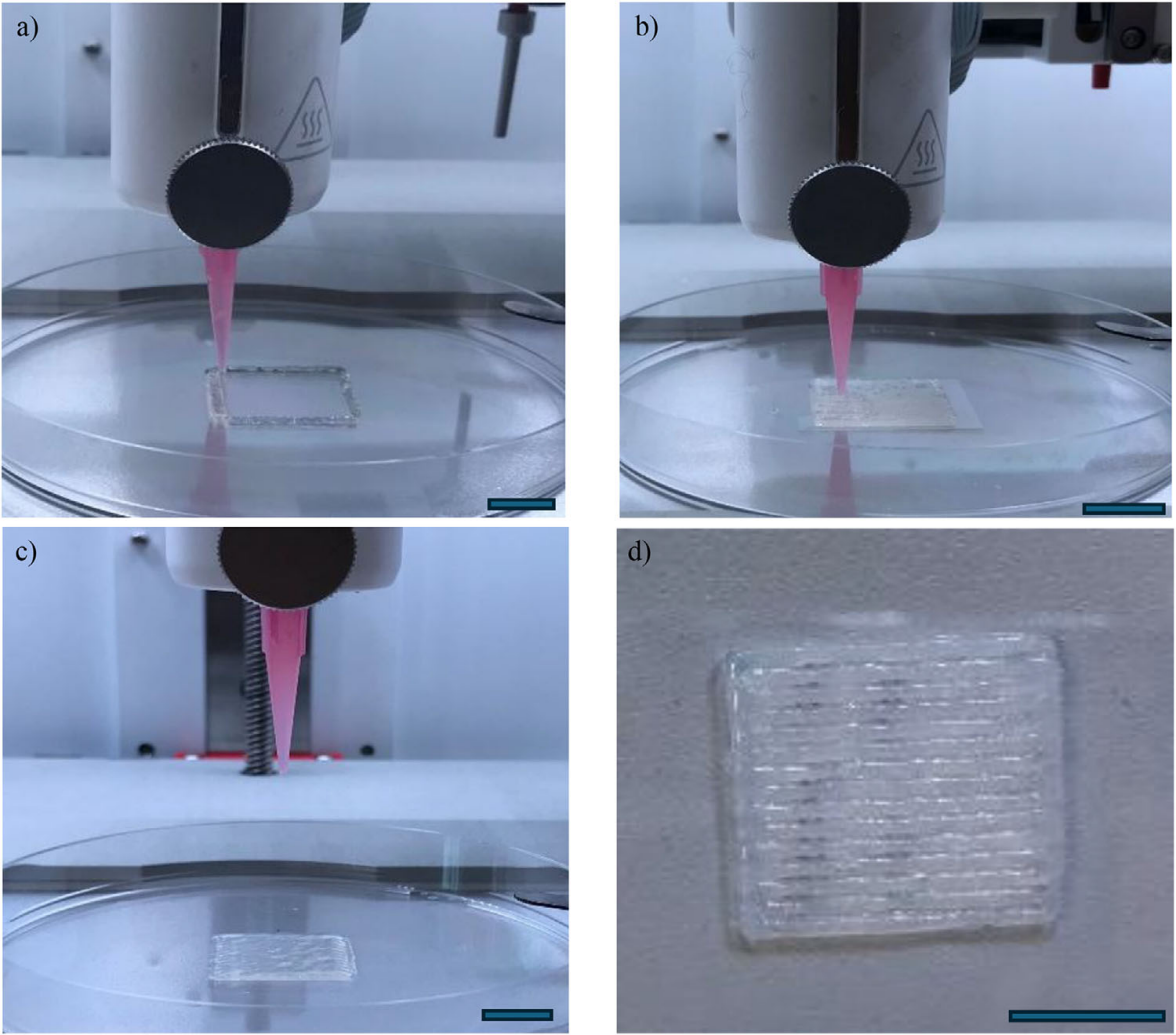
a) First (bottom) layer of 3D ALG‐GEL‐COL constructs printed from gels crosslinked with 0.5% w/v CaCl_2_; b) second (top) layer of 3D ALG‐GEL‐COL constructs bioprinted on top of the first layer from gels crosslinked with 0.5% w/v CaCl_2_; c) the resulting 3D ALG‐GEL‐COL constructs comprising the two printed layers; d) the final two layered 3D bioprinted ALG‐GEL‐COL constructs from closer field of view. Scale bar: 10 mm.

The images shown in Figure 3 summarize the printing steps followed to obtain the cell‐laden 3D constructs. Bioinks based on natural biopolymers such as ALG, COL, GEL, hyaluronic acid, chitosan, fibrinogen and GEL methacrylate are widely used in tissue regeneration applications due to their well‐established biocompatibility and safety profiles [24, 36, 37].

To better mimic the skin's ECM, COL was initially selected as printing gel, however, COL is challenging when used directly in 3D printing due to its poor viscoelastic and mechanical properties. Therefore, combining with other gelation materials such as GEL and ALG in different ratios can help overcome these limitations [38]. When the 3D printed cell‐laden constructs were incubated in culture media at a temperature above room temperature, the GEL readily hydrated, whereas the crosslinked ALG maintained its structural integrity [39]. Previous studies [40] have reported that the highest concentration of COL that can be combined with ALG and GEL for bioprinting to achieve a stable 3D hydrogel network was 10% w/v and this concentration was therefore added to the composite bioinks for printing in our study.

Figure 3d showed that the 3D printed ALG‐GEL‐COL constructs exhibited a clear and stable structure with interconnected channels while possessing smooth and uniform morphology with a thickness of 1 ± 0.2 mm. Further, the printed bioink materials had sufficient structural integrity which allowed self‐support for layer‐by‐layer fabrication and confirmed the suitability for extrusion‐based bioprinting [41]. However, bioprinting via extrusion using a fully crosslinked bioink was not deemed effective due to the very high shear stress required for printing which would be deleterious to cell viability as well as resulting in poor adhesion between the two printed layers. To avoid these challenges, the specified concentrations of the ALG‐GEL‐COL and CaCl_2_ solutions were used to form partially crosslinked ALG based bioinks that possessed a balance between mechanical rigidity and relatively low viscosity to maintain construct integrity while avoiding the use of high shear stresses during printing. Lower ratios resulted in complete crosslinking of the ALG‐GEL‐COL to form non‐homogeneous gels which were not easy to bioprint. Higher ALG‐GEL‐COL: CaCl_2_ ratios produced more homogenous bioinks, however, the rigidity was significantly low and could not form a structure when bioprinted. The suitable volume ratio to partially crosslink the composite gels of ALG‐GEL‐COL:CaCl_2_ was 4:1 which was subsequently exposed to a higher concentration of CaCl_2_ post printing to achieve a fully crosslinked scaffold. This is in line with previously published work [42] where CaCl_2_ was initially applied to partially crosslink and subsequently fully crosslinked to obtain stronger ALG hydrogels.

### 3.3 Degradation Studies

**FIGURE 4:**
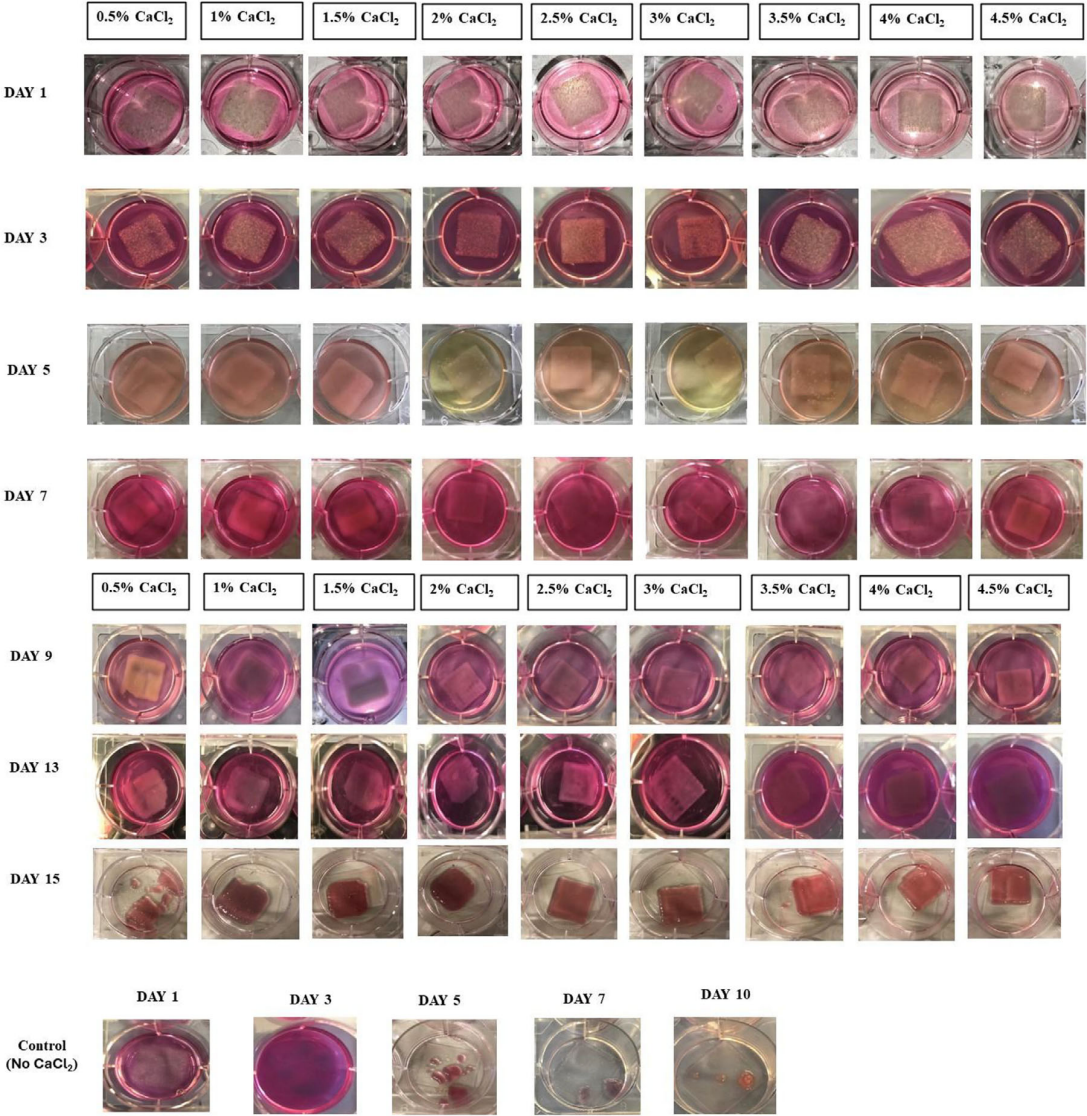
Plain (no HDFs and KCs) ALG‐GEL‐COL 3D bioprinted constructs, exposed to different concentrations of CaCl_2_ (0.5‐4.5% w/v) for 3 min, submerged in DMEM media and transferred into incubator over 14 days. ALG‐GE‐COL 3D bioprinted constructs containing no CaCl_2_ were used as control. The images of the constructs were captured every day, and media refreshed every two days.

A major drawback of bioinks crosslinked with Ca^2+^ such as the composite ALG‐GEL‐COL hydrogels is the limited long‐term stability of the resulting printed constructs under physiological conditions as these structures slowly dissolve due to the exchange of Ca^2+^ with Na^+^ ions and subsequently released into the surrounding physiological media [43]. It is, however, essential for the construct to maintain its structure and shape in the physiological environment long enough for cells to grow into the matrix structure and assume the same structure and shape. To determine the optimum concentration of CaCl_2_ to achieve full crosslinking of the 3D printed ALG‐GEL‐COL constructs (with no cells), they were subjected to degradation testing, and the results are shown in Figure 4. As can be observed from the images, the printed constructs exposed to 0.5 and 1% w/v of CaCl_2_ degraded within two weeks, whilst those exposed to 1.5% and higher concentrations of CaCl_2_ remained intact. Although 3D printed constructs fully crosslinked with 4.5% w/v of CaCl_2_ showed the slowest rate of degradation, these constructs were not selected due to concerns about potential cytotoxicity of high CaCl_2_ concentrations on the KCs and HDFs once embedded within the bioinks. Therefore, 1.5% w/v CaCl_2_ was the selected optimum concentration to fully crosslink the 3D printed constructs. It is important to note that apart from the concentration of the crosslinking agent, the degradation time can be affected by other variables such as printing shape, fiber spacing and total volume of the constructs [40]. Furthermore, it should be noted that the biodegradability test results were only examined visually, which is a drawback of this study. Future studies will require quantitative measurement of biodegradability using well known quantitative methods such as gravimetric studies [44].

### 3.4 Effect of CaCl_2_ on Cell Viability of Bioprinted Constructs

**FIGURE 5:**
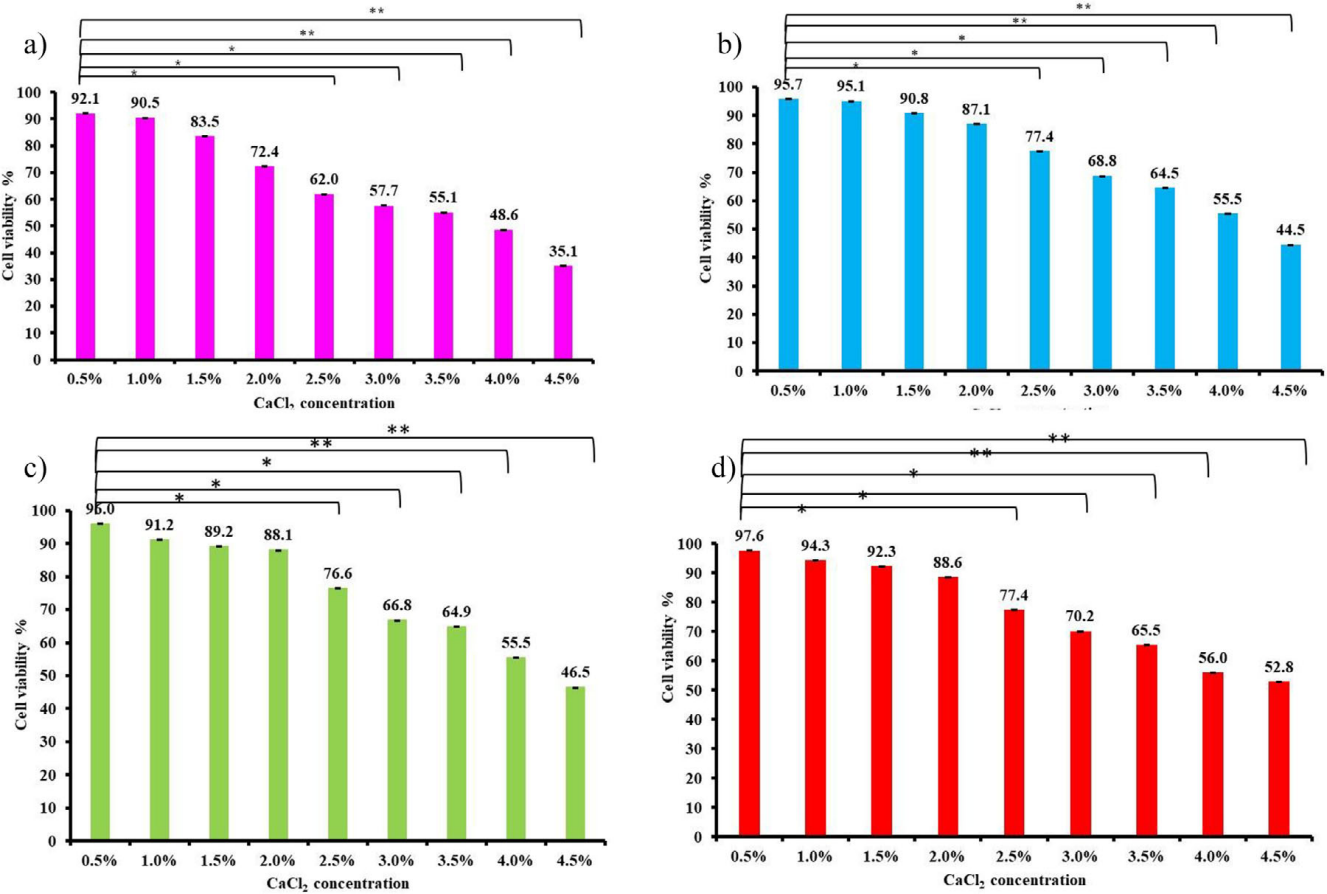
Cell viability profiles obtained by analyzing ALG‐GEL‐COL cell‐laden 3D constructs (having KCs embedded A & B, or HDFs embedded C & D) over 24 h (A & C) and 48 h (B & D) treated with different CaCl_2_ concentrations (% w/v) using MTT assay. The results plotted are shown as means (*n =* 6) of cell viability ± standard deviation. * = p < 0.05; ** = *p* ≤ 0.01.

Figure 5 shows the MTT results of ALG‐GEL‐COL 3D printed constructs containing KCs (A & B) and the constructs containing HDFs (C & D) respectively. The KC laden constructs submerged in 0.5% CaCl_2_ exhibited the highest % viability at 24 h (92%) and 48 h (95%) while those submerged in 4.5% CaCl_2_ showed the lowest % viability at 24 h (44%) and 48 h (35%). Similarly, the constructs embedded with HDFs and submerged in 0.5% CaCl_2_ showed the highest % viability at 24 h (89%) and 48 h (92%) while HDFs laden constructs submerged in 4.5% CaCl_2_ showed the lowest % viability at 24 h (46%) and 48 h (52%). The above values showed that both KCs and HDFs were able to recover from the initial effect of CaCl_2_ as viability increased at 48 h compared to 24 h and also showed that KCs were more sensitive to CaCl_2_ than the HDFs. The ISO guidelines determining biocompatibility of biomaterials and medical devices such as 3D printed constructs specifies a cell viability of ≥70% following exposure as confirmation of their biocompatibility [45]. Based on the degradation studies to determine which 3D printed constructs crosslinked with CaCl_2_ remained intact for two 2 weeks as well as the MTT assay results, the printed constructs crosslinked with 1.5% CaCl_2_ were confirmed as optimum and therefore selected for further analysis.

The ALG‐GEL‐COL cell‐laden 3D printed constructs embedded with a co‐culture of HDFs and KCs and submerged in 1.5% CaCl_2_ were further assessed over a 7‐day period. The KCs/HDFs showed a cell viability of 92% and 96% after 1 and 2 days respectively, while the viability on days 3, 4, 5, 6 and 7 were respectively 97%, 95%, 94%, 98%, and 99%. The results clearly showed that the ALG‐GEL‐COL bioinks are biocompatible and non‐toxic against both HDFs and KCs when in co‐culture, as they consistently demonstrated cell viability values above 90%. This is not surprising since ALG is a naturally occurring, non‐toxic, biodegradable, and non‐immunogenic linear polysaccharide [46] even after crosslinking and widely used as moist wound dressing material clinically. However, crosslinked ALG could also be a hindrance to certain cellular activities and therefore it is expected that the ALG will degrade over time to leave the other biopolymers (GEL and COL). This could be achieved locally through ion exchange between Ca^2+^ crosslinking the ALG and Na^+^ ions present in matrices such as wound exudate and subsequently allows the ALG to dissolve [43]. Interestingly, Vajda and co‐workers [47] designed ALG‐cellulose based matrices encapsulating HDFs and showed that the crosslinked (using CaCl_2_) ALG hydrogels supported high cell viability while the HDFs encapsulated within the matrices were able to retain their original phenotype over a 30‐day period which was confirmed through a steady increase in COL types I and III, deposition of fibronectin as well the expression of specific HDF markers. Furthermore, the impact of the crosslinked ALG on cell activities could be mitigated by the presence of COL and GEL, both of which are known to stimulate cell adhesion, migration, and proliferation [48, 49], with a resultant enhancement in tissue regeneration and wound healing. COL is also highly biocompatible since it is a natural part of the skin matrix, while GEL, which is obtained from hydrolyzed COL, has attracted much attention due to its similarity to human ECM, while possessing good biocompatibility which is advantageous for tissue engineering applications [50]. Wu and co‐authors investigated the viability of human corneal epithelial cells embedded within 3D printed ALG‐GEL‐COL constructs and demonstrated no cytotoxic effect with > 94% cell viability within the first five days after printing [40]. Another interesting observation from the MTT results is that though the ALG‐GEL‐COL bioinks were sterilized using an autoclave (which could potentially have a deleterious effect on the protein structure), the ink still maintained high cell viability following the bioprinting process, suggesting that the protein components (GEL and COL) retained sufficient biological functionality for the intended application. However, further work will be required to directly investigate the effect of autoclaving on the proteins’ structure and any potential impact on the biological performance in vivo as well as using alternative techniques such as UV irradiation or ethylene oxide gas.

### 3.5 Live‐Dead Assay of 3D Printed Constructs

Further advanced cell viability testing was undertaken to distinguish between live cells (stained with calcein green) and the total number of cells (stained with DAPI blue) for both HDFs and KCs in co‐culture. Confocal images demonstrated high cell viability for all the constructs at all‐time points. This applied to (a) constructs comprising two layers loaded with co‐cultures of KCs and HDFs (Figure S2) and (b) the constructs with one layer containing only KCs and the other layer comprising only HDFs (Figure S3). The final cell viability value on day 7 was 96% for constructs with layers laden with the mixed co‐culture of KCs and HDFs (Figure 7), while the final (day 7) cell viability for the constructs with one layer containing only KCs and the other layer containing only HDFs, was 98%.

**FIGURE 6:**
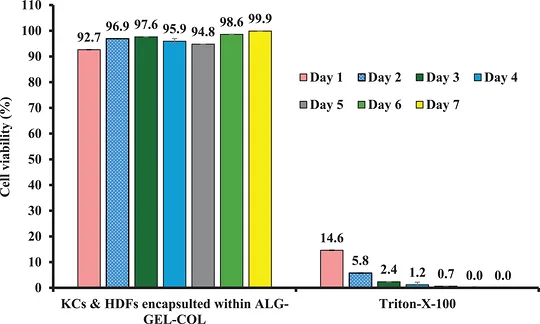
MTT assay cell viability profiles over a 7‐day period for the ALG‐GL‐COL 3D printed constructs embedded with HDFs and KCs, treated with the optimal concentration (1.5% w/v) of CaCl_2_. The plotted results are shown as the mean of six replicates (*n* = 6 ± standard deviation). There were no significant differences observed between the different days for the CaCl_2_ treated constructs, while the control Triton X‐ demonstrated significant deterioration (*p* < 0.05) with time up to 7 days.

**FIGURE 7:**
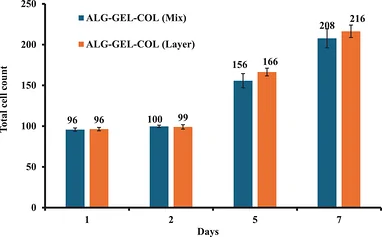
Corresponding total cell counts for the constructs described in Figures S2 and S3 (supplementary information) over the 7‐day period (*n =* 3, ± SD). Differences between experimental data at a level of * *p* < 0.05 were considered statistically significant (days 1 and 2 vs day 5) and that at a level of ** *p* ≤ 0.01 considered highly significant (days 1 and 2 vs day 7).

Both types showed a steady increase in cell viability from days 1 to 7, which is critical to ensure cell development and growth to facilitate skin regeneration following injury. This was achieved by lowering the printing pressure, as the bioink has an optimum viscosity at which the pneumatic pressure can be lowered, to reduce stress on the cells during the printing process. The number of cells increased over the seven days of incubation and showed a doubling from days 1 to 7, for the bioprinted ALG‐GEL‐COL constructs containing co‐cultures of KCs and HDFs in both layers as well as those with only one cell type embedded in each layer. This doubling of cells indicates cell survival and proliferation within the bioink before and after bioprinting (Figure 7) and suggests sufficient permeability of the ALG‐GEL‐COL construct to allow effective diffusion of nutrients and oxygen and subsequent removal of waste. The fact that the embedded cells were metabolically active post printing (Figure 6) and the total cell count increased continuously, indicates cell survival and growth. The increased doubling time may be a consequence of the new culture conditions which is supported within the literature [51] for 3D cultures of these cell types. Further, it shows that the printing materials (ALG, COL, GEL) and parameters used in this study did not have any deleterious effect on the cells present within the bioprinted constructs and that they could sustain cell viability without disruption of the constructs. The long‐term stability of the constructs in the relevant culture medium is an important characteristic for potential applications in tissue regeneration. The incorporation of COL within the bioink for bioprinting did not only better mimic the skin‐specific ECM but also resulted in a more fibrous 3D structure. Rutz and co‐workers noted that such a fibrous structure enabled native skin cells to recognize the construct more readily and enhanced the construct's overall robustness [36]. In a related study by Palladino and co‐workers [52], cell‐laden pure COL scaffolds were 3D bioprinted in the presence of different concentrations of tannic acid as an external crosslinker during bioprinting. Their live‐dead assay results showed that on day 1 post‐bioprinting, the cell viability was low at 45% for scaffolds crosslinked with 20 mg/mL of tannic acid, but this increased significantly over time and reached a high value of 81% on day 7. This demonstrates the ability of COL‐based scaffolds to interact with and stimulate metabolic activity and subsequently enhance cell proliferation and viability over time. In an interesting study, Fu and co [53], produced artificial skin substitute constructs with biomimetic properties. The constructs comprised COL encapsulating HDFs, to enhance the wound healing progression of diabetic wounds through the critical phases. These constructs exhibited very effective antioxidant and anti‐inflammatory action, accelerated COL deposition and subsequent re‐epithelialization. In addition, they enhanced angiogenesis, which prevented hypoxia and development of chronic wounds and consequently ensured effective regeneration of skin tissue.

### 3.6 Investigation of Different Cell Types and Cell Mobility Within the Bioink Using Immunofluorescence (IF) Studies

**FIGURE 8:**
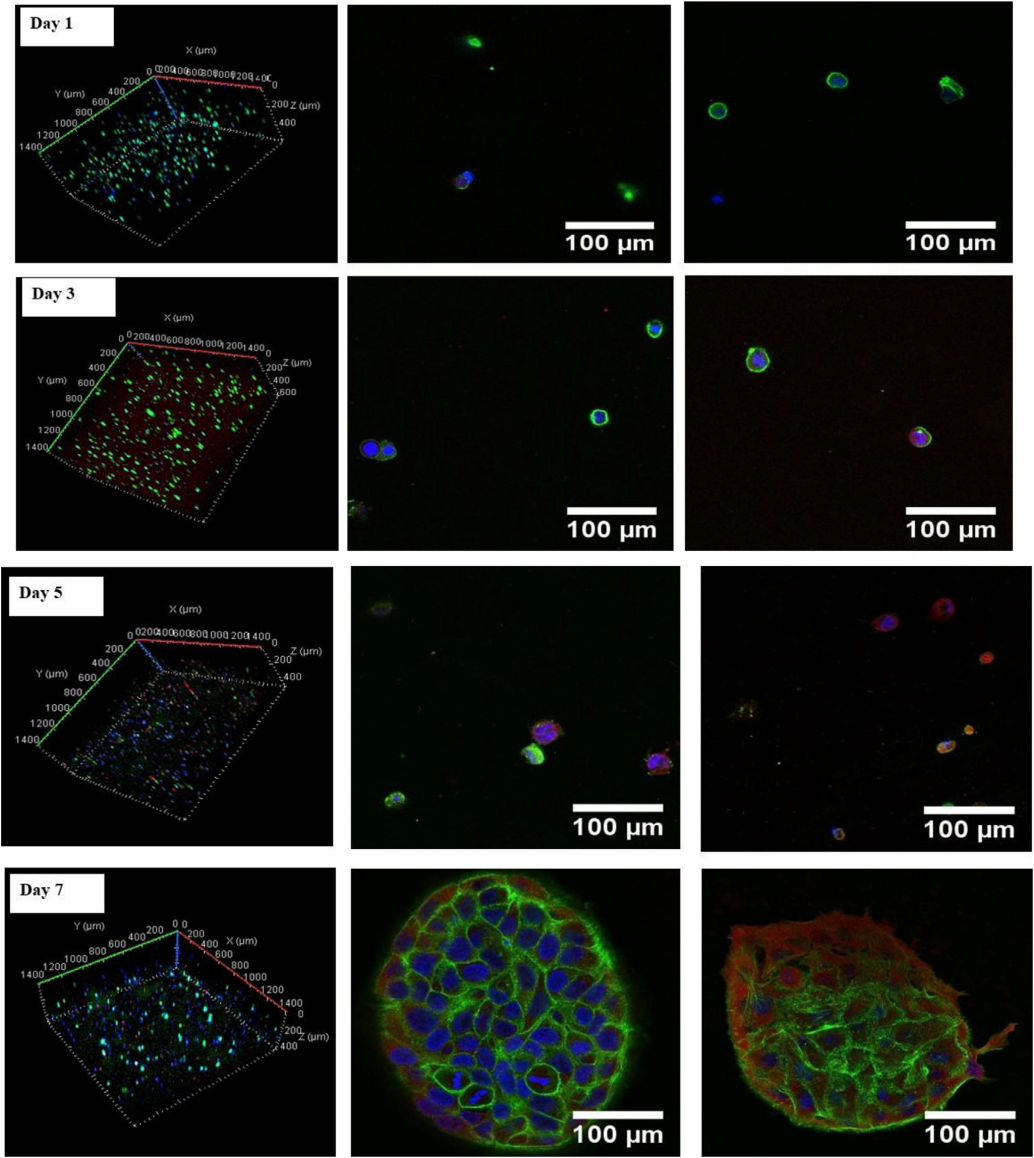
Immunofluorescent images of bioprinted ALG‐GEL‐COL constructs comprising two layers of KCs and HDFs in mixed co‐culture, its projection of pan‐keratin‐containing KCs and FITC phalloidin containing KCs and HDFs over the 7 days. The images show cell nuclei (blue), pan‐keratin containing KCs (red) and FITC phalloidin (green) as actin labelling.

**FIGURE 9:**
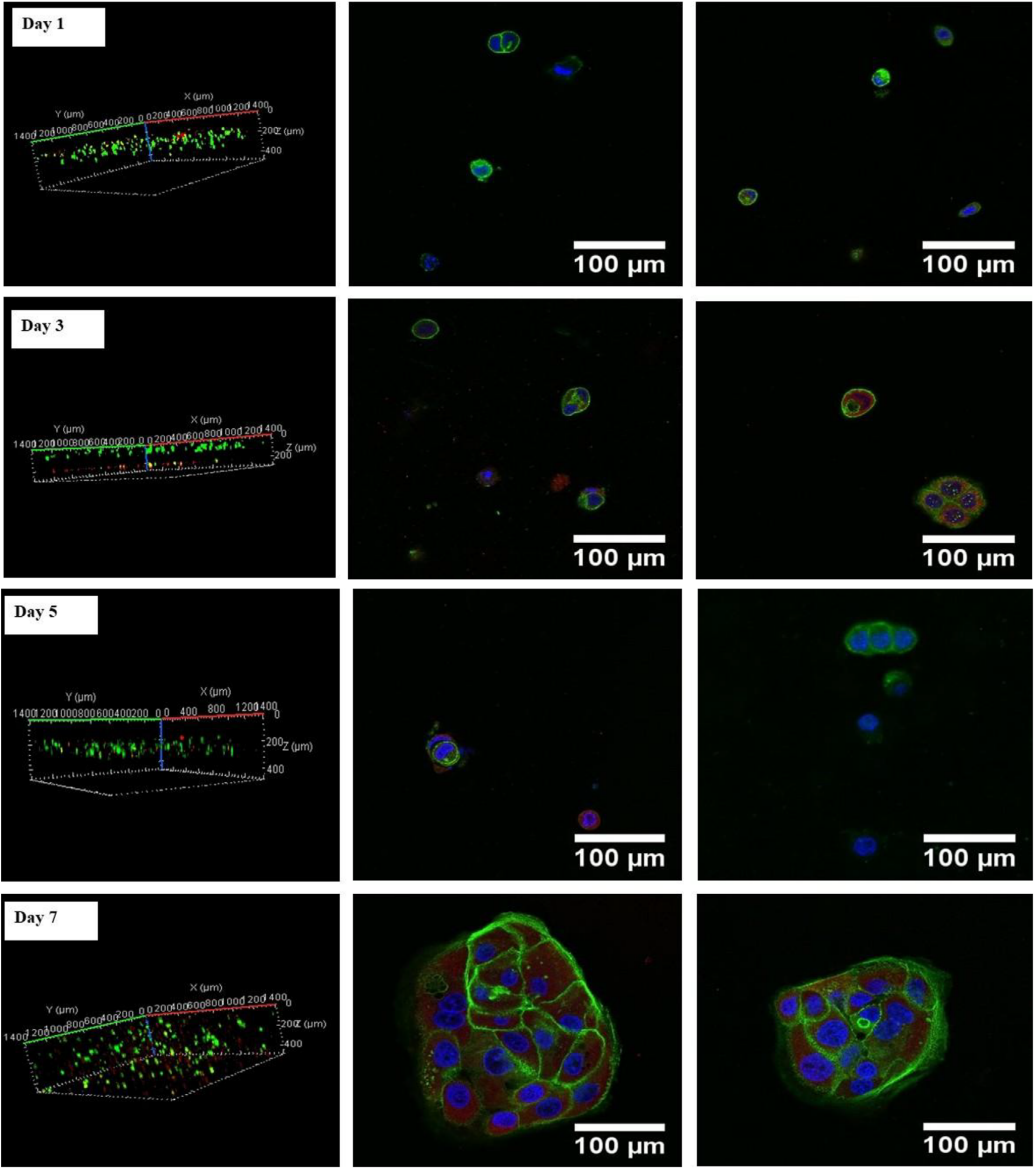
Immunofluorescent images of bioprinted ALG‐GEL‐COL constructs having one layer comprising only the KCs and the other layer containing only the HDFs over 7 days. Nuclei are labelled in blue (DAPI) while keratin and FITC phalloidin are labelled in red and green.

To study cell dynamics, and mobility in the 3D printed constructs, cells printed in a co‐culture and in separate layers were immunostained with pan keratin for KCs and phalloidin for all cells. The reason for bioprinting two layers of KCs and HDFs was to determine whether KCs can migrate to the upper layer and HDFs to the bottom layer and subsequently build layers that mimicked the skin structure. Figures 8 and 9 show confocal microscopy images of the bioprinted ALG‐GEL‐COL constructs over 7 days of culture. Both HDFs and KCs within the mixed co‐culture in the bioprinted construct showed cell growth over the 7‐day period, and both cell types (KC‐ red and green, HDF‐ green) were observed at all time points with good spatial distribution. Cell colonies could be seen starting to form on day 7 of culture, indicating cell growth and metabolic activity and this is supported by Figure 7. In a study by Cavallo et al., [54] bioprinted hydrogel bioink comprising fibrinogen, ALG crosslinked with CaCl_2_ and embedding a dermal layer comprising different cell densities (1 × 10^6^, 2 × 10^6^ and 4 × 10^6^ cells/mL) of HDFs and a top layer comprising KCs (HaCaT cells) were produced to obtain an artificial skin equivalent. Live and dead assay and histological analysis showed tissue‐like constructs exhibiting two separate layers, showing the viable and proliferating cells in both layers. The cell morphology results showed that cell proliferation could not be observed for constructs bioprinted from inks comprising 1 and 2 × 10^6^ HDF cells/mL, though changes in cell morphology were observed for bioinks comprising 2 × 10^6^ HDF cell/mL. After 7 days, a small number of spreading cells were observed within the dermal layer, while cell clusters exhibiting HDF characteristic morphology were visible on day 14. At 4 × 10^6^ HDF cells/mL of bioink live cells within the within the constructs were visible with the shape of the cells changing on day 7, as well as demonstrating excellent cell proliferation.

Cells printed in distinct layers (Figure 9) demonstrated that both bottom and upper cell layers contained FITC phalloidin with a distinctive layer of KC visible by day 3 (KC‐ red and green, HDF‐ green). As time progressed over the 7 days, it was observed that the cell expanded through the Z axis (Figure 8, day 7) where cells can be seen through the whole Z section, compared to day 3 where cells were observed in two thinner more distinct cell layers. This indicates cell motility through the gel with both KCs and HDFs visible through the 3D structure of the mixed culture by day 7. This suggests that cell‐laden ALG‐GEL‐COL constructs containing KCs and HDFs in separate layers can migrate through the printed construct, indicating that printing KCs and HDFs in separate layers is potentially suitable for building a skin layer. Cell morphology was non‐standard in both the printed layers and co‐culture, with both HDFs and KC having a round morphology (Figures 8 and 9) which implies that the bioink is low‐adhesive, and the cells did not fully adhere to the bioink. The rounded morphology of the HDFs and KCs implies they are in an unspread and suspended state which is typical of cells during division or prior to cell adhesion. Temporarily maintaining a round morphology is of critical importance for cell differentiation re‐programming to form other cell types [55] in the case of HDFs, as well as for engineering of cellular function for example in clinical grafting [56] and re‐epithelialization in the case of KCs.

For example, Wang and co‐workers [57] showed that KCs with rounded cell morphology at the wound margins migrate and communicate with HDFs in the presence of growth factors such as keratinocyte growth factor and heparin‐binding epidermal growth factor to facilitate closure of the wound. ALG has non‐adhesive properties for cell attachment and therefore the cell morphology observed is to be expected. Although this bioink had low cell adhesive properties, it could still maintain cell growth and motility and was therefore deemed a suitable bioink for promoting skin tissue self‐assembly and creating more physiologically relevant 3D models [58].

## 4 Conclusion

This study evaluated composite bioinks comprising three natural biopolymers, ALG, GEL, and COL for 3D bioprinting to achieve 3D constructs embedding HDFs and KCs to mimic human epidermal/dermal skin layer. The optimum bioink with desirable rheological properties for extrusion based bioprinting was determined to be a composite mixture comprising equal volumes of 6% w/v gel solutions for both ALG and GEL and 10% w/v gel solution for COL. This optimum blend of ALG‐GEL‐COL matched the rheological profiles of expensive commercial bioinks available in the market for skin bioprinting, therefore providing an alternative cheaper in‐house bioink for skin regeneration studies. The composite ALG‐GEL‐COL bioink and the optimized printing process settings were mild on the embedded HDFs and KCs resulting in easy printing of these cell types through an extrusion‐based 3D bioprinting with no deleterious effects on the cells. The ALG‐GEL‐COL bioink exhibited sufficiently high viscosity ideal for HDF and KC cells, the 3D printed construct remained stable in the presence of physiological media over 14 days, while the minimization of shear stresses during bioprinting resulted in high cell viability being achieved post printing. The immunofluorescence confocal microscopy images confirmed the presence of both KCs and HDFs in the 3D bioprinted constructs. In addition, the formation of a KC layer on top of the bioprinted construct indicates the capability to bioprint highly viable KCs through fully automated extrusion bioprinting. The results demonstrated the suitability of ALG‐GEL‐COL for future skin regeneration studies, but additional histological characterization is required over a longer time period to confirm the formation of full thickness epidermal and dermal skin layers.

## Conflicts of Interest

The authors declare no conflicts of interest.

## Supporting information




**Supporting File**: mabi70253‐sup‐0001‐SuppMat.docx.

## Data Availability

The data that supports the findings of this study are available in the supplementary material of this article.
