# Supplementary material for: 3D Bioprinted Alginate‐Gelatin‐Collagen Based Hydrogels Laden With Fibroblast and Keratinocyte Cells Show Potential for Regeneration of Injured Skin
Source: Macromol Biosci. 2026 Sep 9;26(9):e70253. doi: 10.1002/mabi.70253 (PMC13556172; doi:10.1002/mabi.70253)
Supplement: Supplementary file 1 [file MABI-26-e70253-s001.docx]

SUPPLEMENTARY INFORMATION

# **3D Bioprinted Alginate-Gelatin-Collagen Based Hydrogels Laden with Fibroblast and Keratinocyte Cells Show Potential for Regeneration of Injured Skin**

Joshua Boateng^1^*, Forough Hafezi^1^, Susan Shorter^1^, Atabak Tabriz^1^, Dennis Douroumis^1^

^1^Faculty of Engineering and Science, University of Greenwich, Medway, Central Avenue, Chatham Maritime, Kent ME4 4TB.

* Correspondence: [j.s.boateng@gre.ac.uk;joshboat40@gmail.com](mailto:j.s.boateng@gre.ac.uk;joshboat40@gmail.com)

**S1: Bioprinting design**

Table S1 Bioprinting process parameters for the designed bioinks.

| **Bioinks** | **Pressure (Kpa)** | **Speed (mm/s)** | **Infill density (%)** |
| --- | --- | --- | --- |
| **ALG-GEL-COL (0.2-1.2%w/v CaCl_2_)** | 5-15 | 4-6 | 30-50 |
| **Cellink start** | 28 | 5 | 35 |

**S2 Autoclaving of partially crosslinked bioinks**

**Before autoclaving**

**After autoclaving**

Figure S1 Digital images of the partially crosslinked ALG-GEL-COL bioink before and after sterilization using autoclaving

**S3 Confocal microscopy**

Day 7

Day 1

Day 5

Day 3

Day 3

Figure S2 Confocal images of bioprinted ALG-GEL-COL bilayer constructs with each layer laden with co-culture of both KCs and HDFs over seven days showing live (green) and total number of the cells (blue). All images shown in this graph are representative of 3 independent experiments.

Day 3

Day 1

Day 7

Day 5

Figure S3 Confocal images of bioprinted ALG-GEL-COL constructs with one layer containing only KCs and the other layer comprising only HDFs over a period of seven days showing live (green) and total number of the cells (blue). The images shown are representative of 3 independent experiments.
